# The number of α-synuclein proteins per vesicle gives insights into its physiological function

**DOI:** 10.1038/srep30658

**Published:** 2016-08-01

**Authors:** Mohammad A. A. Fakhree, Niels Zijlstra, Christian C. Raiss, Carolus J. Siero, Heinrich Grabmayr, Andreas R. Bausch, Christian Blum, Mireille M. A. E. Claessens

**Affiliations:** 1 Nanobiophysics Group, MIRA and MESA+ Institutes, University of Twente, Enschede, the Netherlands; 2 Lehrstuhl für Biophysik (E27), Technische Universität München, 85748 Garching, Germany

## Abstract

Although it is well established that the protein α-synuclein (αS) plays an important role in Parkinson’s disease, its physiological function remains largely unknown. It has been reported to bind membranes and to play a role in membrane remodeling processes. The mechanism by which αS remodels membranes is still debated; it may either affect its physical properties or act as a chaperone for other membrane associated proteins. To obtain insight into the role of αS in membrane remodeling we investigated the number of αS proteins associated with single small vesicles in a neuronal cell model. Using single-molecule microscopy and photo-bleaching approaches, we most frequently found 70 αS-GFPs per vesicle. Although this number is high enough to modulate physical membrane properties, it is also strikingly similar to the number of synaptobrevins, a putative interaction partner of αS, per vesicle. We therefore hypothesize a dual, synergistic role for αS in membrane remodeling.

α-Synuclein (αS) is an intrinsically disordered protein of 14.3 kD that is abundant in the brain where it represents a considerable part of the cytosolic protein content^1^. The protein αS has been suggested to play a key role in the development of Parkinson’s disease (PD), a neurodegenerative disorder associated with loss of dopaminergic neurons in the substantia nigra^2^. Although the involvement of αS in PD pathology is well established, the exact physiological role of this protein remains an enigma.

αS is enriched in the presynaptic termini of dopaminergic neurons. At these termini the soluble protein is in equilibrium with vesicle bound αS^3^. Membrane bound αS has been suggested to interact with other synaptic vesicle proteins. It has been reported to chaperone the formation of the SNARE complexes^4^,^5^,^6^ and thereby mediate membrane fusion processes. *In vitro* experiments hint that, at high protein to lipid ratios, αS can also directly change the physico-chemical membrane properties. From *in vitro* experiments, αS has been reported to increase lipid packing^7^ and induce positive mean and negative Gaussian curvatures in phospholipid bilayers^8^. Additionally, αS binding has been observed to cause tubulation and fragmentation of phospholipid vesicles at high αS concentrations^9^,^10^,^11^, and reduce membrane tension and increase undulations in small unilamellar vesicles^12^. Although these changes in membrane properties agree well with a role for the protein in membrane remodeling processes, it is still unclear if the changes in membrane properties observed *in vitro* are relevant *in vivo*.

The two suggested functions of αS – being an interaction partner for other proteins or changing the physico-chemical properties of the membrane – require distinctly different numbers of αS per vesicle. Whereas for specific interactions with synaptic vesicle proteins only one or a few membrane bound αS molecules may be needed, larger surface concentrations are required if the physiological function of the αS involves changing physico-chemical properties of the membrane. The copy number of most synaptic vesicle proteins is low. Interestingly, αS is absent in reports on the copy number of major protein constituents of synaptic vesicles^13^. This absence might result from the equilibrium association of αS with membranes and the loss of bound protein during synaptic vesicle purification.

Here, we investigated the number of αS per vesicle in cells, thereby avoiding complications arising from vesicle purification. Using photo-bleaching experiments on αS-GFP expressing cells, we find a most frequent number of 70 αS-GFPs per vesicle. We discuss the observed surface concentration in relation to the possible physiological function of the protein.

## Results

**Figure 1:**
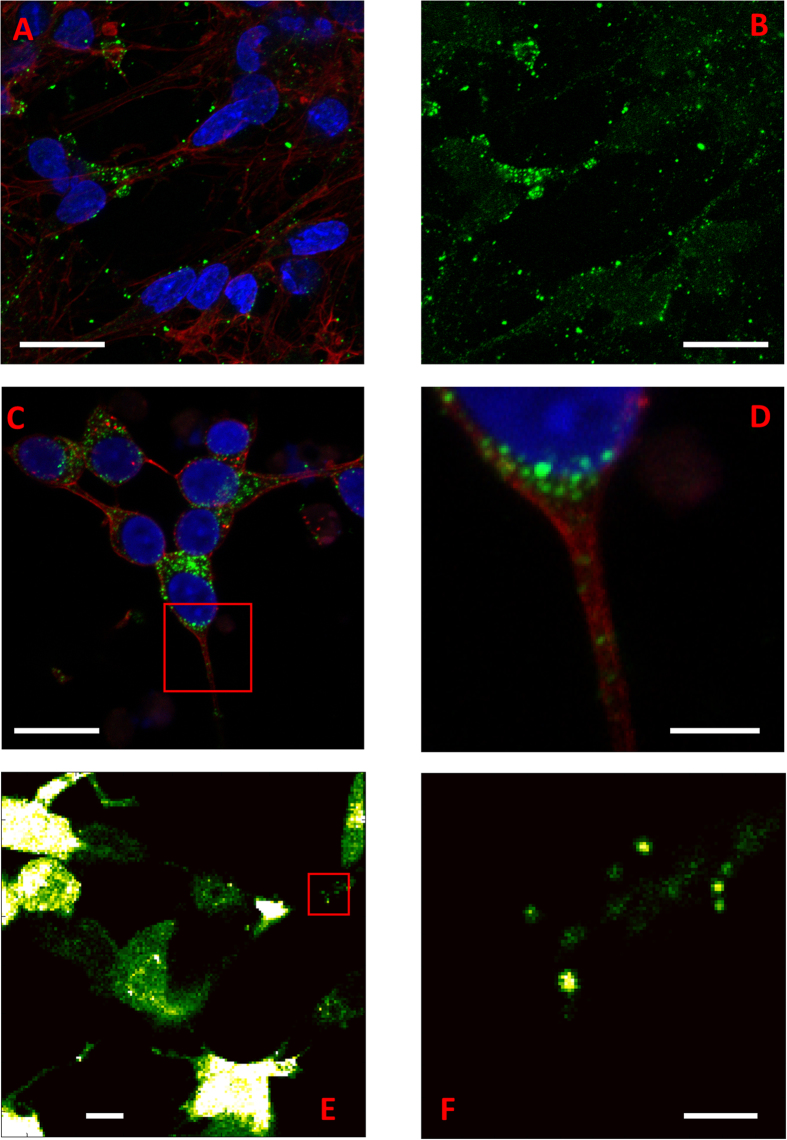
αS distribution in primary neurons and differentiated SH-SY5Y cells. (**A**) Confocal microscopy image of rat primary neurons immunostained for αS (green), actin filaments (red), and nuclei (blue). (scale bar is 20 μm). (**B**) Green channel of (**A**) showing immunostaining for αS. Diffuse signal throughout the neurons and distinct bright αS puncta are visible throughout the neurites and soma of the neurons (scale bar is 20 μm). (**C**) Confocal microscopy image of differentiated SH-SY5Y cells immunostained for αS (green), actin filaments (red), and nuclei (blue). As expected for a neuronal cell model system, the similarities to primary neurons (see panel (**A**)) are evident. (scale bar is 20 μm). (**D**) Zoom of the area indicated with the red square in (**C**) Isolated puncta of αS can be observed in the cell cytoplasm and extensions. (scale bar is 5 μm). (**E**) Single-molecule confocal microscopy image of αS-GFP signal in differentiated SH-SY5Y cells. The fluorescence is not homogenously distributed throughout the cells. In both the cytoplasm and the extensions, high intensity αS-GFP puncta can be observed. (scale bar is 10 μm). (**F**) Zoom of the area indicated in (**E**) Isolated puncta of αS-GFP can be observed in the cell extension. (scale bar is 1 μm).

In images of primary neurons immunostained against αS, two distinct types of fluorescence signals are visible: a diffuse signal spread throughout the cell, and localized, small, high intensity αS puncta (Fig. 1A,B). In these neuronal cells, the αS puncta possibly represent synaptic or endocytic vesicles^3^,^14^. The radius of synaptic vesicles has been reported to range from 15 to 37 nm with an average of 21 nm^13^, whereas endocytic vesicles in the synapse of neuronal cells have a typical radius of 40 nm^15^. Clearly, these sizes are below the resolution limit of conventional optical microscopy, which is roughly 250 nm in radius. Hence, synaptic vesicles appear as diffraction limited sized features in the microscopy images.

Although immunostaining gives excellent results in visualizing and localizing αS in primary neurons, we not only aim to visualize αS, but also to determine the number of αS per vesicle. Unknown and varying labeling efficiencies plus possible steric hindrance due to the size of the antibodies preventing occupation of all possible binding sites, could easily lead to incomplete labeling of membrane bound αS which makes immunostaining unsuitable for quantitative studies.

Single molecule detection based techniques are by now well established in molecular life science^16^,^17^,^18^,^19^,^20^. To determine the number of molecules in complexes or molecular aggregates, single molecule photo-bleaching of fluorescent labels has been shown to be a viable technique^21^,^22^,^23^,^24^. In a cellular context, these photo bleaching based techniques require recombinant labeling of the protein of interest. These strategies, primarily based on GFP labeling of αS, have been proven successful before even in a mouse model of Parkinson’s disease^3^. A control experiment verifies that the GFP tag does not change the interaction between αS and membranes. Circular dichroism experiments show that αS and αS-GFP bind phospholipid vesicles with comparable affinity (See Supplementary Fig. 1), which is in agreement with literature^25^. Therefore, we used αS tagged C terminally with GFP (αS-GFP) and expressed it in a SH-SY5Y cell model. The SH-SY5Y cell model has been shown to be, after differentiation, a suitable cell model system for neurons in neurodegenerative diseases like PD^26^,^27^.

Indeed we find close resemblance between immunostained primary neurons (Fig. 1A,B) and immunostained differentiated SH-SY5Y cells (Fig. 1C,D). In all cell types we find both a diffuse distribution of αS and a localization of αS in well-defined puncta. For the photo-bleaching experiments, we subsequently expressed αS-GFP in the SH-SY5Y cell model system (See Supplementary Fig. 2).

Since our study is based on the observation of bleaching steps from single GFP molecules, the use of ultrasensitive single-molecule fluorescence spectroscopy is required. In Fig. 1E,F we present typical images of our αS-GFP expressing cell line using ultrasensitive microscopy. We observed both a diffuse αS-GFP signal and a spotted pattern of αS puncta throughout the differentiated cells. The observation of both a diffuse signal and localized puncta agrees well with the observations from immunostained primary neurons and immunostained differentiated SH-SY5Y cells (Fig. 1A–D). We attribute the diffuse background signal to cytosolic αS-GFP or αS-GFP associated with vesicles that cannot be resolved individually. The small, high intensity puncta are within the size range expected for diffraction limited structures and might hence represent αS-GFP associated with synaptic and/or endocytic vesicles. Since the size of these puncta cannot be further resolved with conventional confocal fluorescence microscopy, we used STED super-resolution microcopy. From STED microscopy images (see Supplementary Fig. 3), we conclude that the structures giving rise to the puncta in our differentiated SH-SY5Y expressing αS-GFP model cell system are smaller than 80 nm, the resolution limit of the used STED setup, and are hence small enough to represent synaptic or endocytic vesicles.

To confirm that the puncta are indeed vesicles, we subsequently investigated the co-localization of the fluorescent αS-GFP puncta with membranes. To label membranes, we used the membrane marker wheat germ agglutinin, tagged with the fluorophore Alexa Fluor^®^647 (WGA-AF647). The GFP and AF647 fluorophores can be excited and detected independently and by overlaying the images, co-localization can be visualized. To show co-localization of αS-GFP puncta with the membrane, WGA was incubated with the cells for six hours. Independent excitation and detection of GFP (excitation 485 nm, detection 550/88 nm) and WGA (excitation 640 nm, detection >665 nm) resulted in co-localization of 40% of αS-GFP puncta with WGA-AF647 (see Methods).

In our co-localization experiments, upon excitation of GFP, we detected more intense emission above 665 nm than expected from direct excitation of AF647 by 485 nm laser light, and leakage of GFP emission into the detection window beyond 665 nm. Förster Resonance Energy Transfer (FRET) between the initially excited αS-GFP and WGA-AF647 could be the reason for this observation. When the two fluorescent markers, GFP and AF647, are in nanometer proximity, they form a FRET system in which the GFP (donor) fluorescence is efficiently quenched by the presence of AF647 (acceptor). The characteristic Förster distance for the GFP and AF647 pair is expected to be slightly smaller than the 5.6 nm reported for the spectroscopically similar AF488 and AF647 pair. This smaller distance results from the smaller quantum efficiency of GFP compared to AF488.

**Figure 2:**
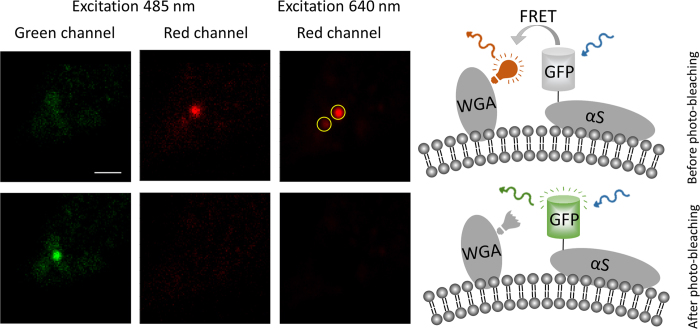
FRET quenching of GFP emission by AF647. As long as the acceptor of the FRET pair (AF647 on WGA) is in close proximity to the donor (GFP on αS), there is no emission from the donor GFP, upper panels, before photo-bleaching. Photo-bleaching of AF647 removes the FRET acceptor, and energy can no longer be transferred away from GFP which results in dequenching of GFP, lower panels, after photo-bleaching. The scale bar is 2 μm and refers to all images. The photo-bleaching experiments were done on puncta which appeared only in red channel upon 485 nm excitation.

Moreover, in our experiments, a large fraction of puncta appeared only in the red channel (emission >665 nm, emission of AF647) after GFP excitation at 485 nm, indicating very efficient FRET between αS-GFP and WGA-AF647 (Fig. 2, top panel). Furthermore, after photo-bleaching the FRET acceptor AF647 with high intensity laser light of 640 nm, the emission of the FRET donor, αS-GFP, appeared in the green channel (emission detection 550/88 nm)(Fig. 2, low panels). After this acceptor bleaching of AF647, and resulting dequenching of αS-GFP, co-localization between αS-GFP and WGA-AF647 increased from 40% to 60%. The formation of such an efficient FRET system implies nanometer proximity between αS-GFP and WGA-AF647. This nanometer distance could result either from a direct interaction between WGA and αS or GFP, or juxta positioning due to high local concentrations. FRET experiments on solutions of mixtures of WGA and αS or GFP excluded relevant direct interaction between WGA and αS or GFP (see Supplementary Fig. 4), suggesting that a high local density of αS-GFP near the membrane marker is most likely responsible for the observed FRET. Furthermore, nanometer proximity between αS-GFP and the membrane bound WGA-AF647 indicates that αS-GFP is in all likeliness membrane bound. This conclusion is further supported by the observation that in some larger vesicular structures, αS-GFP outlines the vesicle contour (see Supplementary Fig. 5). We hence conclude that the observed fluorescent puncta indeed represent vesicle bound αS-GFPs.

After establishing that the observed fluorescent puncta in the studied model cells originate from vesicle bound αS-GFP, the number of αS-GFPs per vesicle was determined. Recently, a number of methods have been developed that use single-molecule photo-bleaching to determine the number of fluorescently labeled subunits in a molecular complex or aggregate^21^,^22^,^23^. For larger complexes, *Leake et al.* pioneered an elegant method to determine the number of labeled subunits per complex that is suitable for *in vivo* quantification^24^. The number of fluorophores in a complex is determined by dividing the background subtracted initial fluorescence intensity by the average intensity per fluorophore, which is determined using a statistical analysis of each bleaching trace (see Methods). To confirm the applicability of this approach to our experimental system with small cellular vesicles, we tested the approach on unilamellar vesicles (UVs) with an average radius of 50 nm and a known average number of membrane embedded fluorophores. We then experimentally determined the number of fluorophores per UV using single-molecule photo-bleaching and the analysis method described above. We find that the determined and expected values of fluorophores per UV agree very well (see Supplementary Fig. 6). Hence, our control experiment verifies the suitability of the method to determine the number of fluorophores per vesicle.

**Figure 3:**
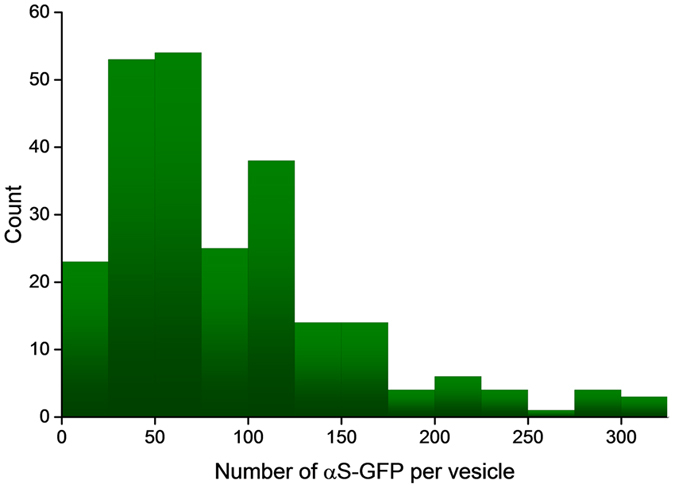
Number of αS-GFPs per vesicle. Distribution of the number of αS-GFPs per vesicle in differentiated SH-SY5Y cells determined from photo-bleaching experiments (N = 243). To minimize the influence of sampling more than one vesicle, puncta with a radius of less than 350 nm in the fluorescence image were selected from the thin parts of the cells. We find a distribution of the number of αS-GFPs per vesicle with a most frequent occurrence of ~70 αS-GFPs per vesicle.

To determine the number of αS-GFP per vesicle in our cell model, we recorded and analyzed bleaching traces obtained from more than 200 distinct puncta, a statistically relevant number. For our study we considered only αS-GFP puncta in the thin parts of the cell (Fig. 1F) with a radius smaller than 350 nm in fluorescence images (see Supplementary Fig. 7 for size distribution). The cutoff size reflects the punctum size expected from the convolution of the physical size of a vesicle with the microscopes point spread function. By using this cutoff criterion we exclude a bias in our data resulting from bleaching traces from large vesicular structures or the presence of multiple small vesicles in the sampled volume. After selecting an αS-GFP punctum that fitted these criteria, high intensity laser excitation (485 nm, 800 W/cm^2^) was used to completely photo-bleach the αS-GFP emission, while recording the fluorescence intensity over time (see example in Supplementary Fig. 8A). The number of αS-GFPs per vesicle was determined from the bleaching traces of 243 distinct vesicles, the copy number was determined and assembled in the histogram presented in Fig. 3.

The distribution of the number of αS-GFPs on the studied vesicles is broad, ranging from around 20 αS-GFPs per vesicle up to more than 300 αS-GFPs, with a most frequent value of approximately 70 αS-GFPs per vesicle. Finding a distribution of the number of αS-GFP per vesicles rather than a defined number matches expectations, since it is well known that the size distribution of vesicles in cells is polydisperse^13^,^28^,^29^. With increasing vesicle surface area the number of αS-GFPs per vesicle is also expected to increase. Interestingly, the shape of the vesicle surface area distribution obtained from the size distribution of synaptic vesicles^13^ and the distribution of the number of αS-GFPs per vesicle we determined, agrees very well. Copy numbers on the high end of the distribution might originate from more than one vesicle in the observation volume, in spite of our size selection criterion.

Using the reported sizes of synaptic vesicles and the number of αS-GFPs per vesicle obtained from the photo-bleaching experiments, it is possible to determine the average distance between αS-GFPs on a vesicle. For the most frequent value of 70 αS-GFPs on an average synaptic vesicle of 21 nm in radius, and neglecting multimerization or co-localization with other proteins, we find an average center-to-center distance of 10 nm between αS-GFPs on the vesicles.

This average distance agrees well with our observation of efficient FRET between GFP and AF647 for some of the vesicles (see Fig. 2). Considering the stochastic nature of both the binding of αS-GFP and the membrane marker WGA-AF647 to the vesicle, variations in energy transfer between αS-GFP and WGA-AF647 per vesicle are to be expected at this average αS density. For relatively few αS-GFPs per vesicle, the FRET partners are spaced further apart than the characteristic Förster distance. Hence for these low copy numbers co-localization of αS-GFP and WGA-AF647 is not accompanied by efficient energy transfer (i.e. 40% co-localization before photo-bleaching of the acceptor). For higher copy numbers, the distances between αS-GFP and WGA-AF647 are below the Förster distance, resulting in quenching of the FRET donor. The higher copy numbers therefore result in an underestimation of the co-localization of αS-GFP and WGA-AF647 (i.e. an increase from 40 to 60% co-localization after photo-bleaching of the acceptor).

## Discussion

We observed diffraction limited fluorescent puncta in a neuronal cell model system of differentiated SH-SY5Y cells expressing αS-GFP. We used STED microscopy and co-localization of the αS-GFP puncta with the membrane marker WGA-AF647 to show that these puncta are vesicles. Efficient FRET between GFP and the fluorescent label AF647 attached to WGA strongly indicates that αS-GFP is membrane bound, which is further supported by our observation that αS-GFP outlines larger membrane structures. We hence conclude that the αS-GFP puncta we observe in our neuronal cell model, are small vesicles with membrane bound αS. We used single-molecule photo-bleaching to determine the number of αS-GFPs per vesicle in the cells. We find a distribution of copy numbers per vesicle with a most frequent number of 70 αS-GFP per vesicle.

We exclude the possibility that the puncta represent the well-defined oligomeric species and amyloid fibrils that have been discussed in the context of Parkinson’s disease^30^,^31^,^32^,^33^. For example, the aggregation number of αS oligomers formed, *in vitro*, under different conditions was determined using single-molecule photo-bleaching experiments^22^,^23^. These *in vitro* formed oligomers typically consist of 30 ± 5 monomers^22^,^34^. Any fibrillar form of αS is expected to contain thousands of αS monomers. It can, however, not be excluded that vesicle bound αS is present as multimeric species.

The distribution in copy numbers reflects the distribution of vesicle sizes and the stochastic nature of binding of αS-GFP to vesicles. Considering that the SH-SY5Y cells were differentiated to neuron like cells, we assume that the studied vesicles are synaptic vesicles. The most frequent copy number of 70 αS-GFPs per synaptic vesicle, is rather high compared to the copy numbers found for most other synaptic vesicle proteins which are in the range of 1 to 10 per vesicle^13^,^35^. In this regard, since the number of αS per vesicle is high, the absence of αS in listings of the copy number of the major protein constituent of synaptic vesicles^13^ is most likely caused by the equilibrium nature of the association of αS with membranes and detaching of αS from the vesicles during vesicle purification. Interestingly, 70 αS per vesicle is a high enough number to alter the physical membrane properties^8^.

In the literature, αS has been reported to change the physical properties of phospholipid bilayers^8^,^9^,^10^. Based on experimental data and simulations, αS has been suggested to be involved in membrane curvature induction and binding of αS has been shown to cause tubulation and vesiculation of lipid bilayers^8^,^9^,^10^. At high molar ratios of protein to lipid; 1:10 to 1:40, αS induces vesiculation and tubulation in POPG lipid bilayers^10^. At lower molar ratios of 1:200, αS induces positive mean curvature and local negative Gaussian curvature in flat lipid bilayers^8^. At this lower protein to lipid ratio, αS was reported to lower the membrane surface tension and increase membrane undulations in small unilamellar vesicles by decreasing the negative pressure in the headgroup region of the outer membrane leaflet and increasing the positive pressure throughout the hydrocarbon core^12^. If synaptic or endocytic vesicles in neurons are stressed by high curvature because of their size, the effect of αS binding on surface tension could stabilize the vesicles and prevent fusion.

Assuming that the observed puncta are synaptic vesicles with an average radius of 21 nm, the average copy number of 70 would result in a molar ratio of 1:250 between αS and lipids. Our data thus supports the idea that membrane binding of αS and the concomitant partial insertion of amphipathic α-helices can stabilize the membrane curvature and control fusion processes of vesicles with a radius smaller than 40 nm. Besides α-helix insertion, the large hydrodynamic radius of the charged unstructured C-terminal domain of the protein may contribute to the promotion of membrane curvature by generating steric pressure^36^. Structural studies of αS bound to sodium dodecyl sulfate micelles show that the protein forms two antiparallel α-helices comprising residues 3–37 and 45–92, while residues 93–140 remain unstructured and are exposed to the solution^37^,^38^. Assuming that the 48 residues at the C-terminus can adopt a free random walk with a persistence length *l*_*p*_ corresponding to roughly 3 amino acids, they would form a globule with a radius of gyration of approximately 
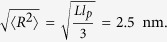
 Using the known average size of synaptic vesicles, we determined the average distance between αS-GFPs on the vesicle surface to be 10 nm. We therefore conclude that αS binding to the already crowded surface of synaptic vesicles may contribute to steric pressure. However, since we used a photo-bleaching approach to determine the copy number of αS-GFP per vesicle, we do not sample endogenous αS. To estimate the ratio between αS and αS-GFP inside cells, we performed a Western blot. Analysis of Western blots of cell lysate indicated that the ratio between the endogenous αS and αS-GFP in the differentiated SH-SY5Y cells was approximately 1:2 (see Supplementary Fig. 2). Since αS and αS-GFP bind membranes with comparable affinity we conclude that the total number of αS proteins, including both αS and αS-GFP, associated with a single vesicle can be as much as 100. Insertion of the helical domain of αS and possibly, in conjunction with other membrane bound proteins, steric pressure exerted by the unstructured αS domain may thus considerably contribute to establishing and maintaining membrane curvature.

As mentioned earlier, αS has been reported to interact with other proteins. These reported interaction partners of αS include a variety of proteins, including, but not limited to the cytoskeletal protein tau^39^, the chaperone proteins HSP70 40, and the SNARE protein synaptobrevin^6^,^41^,^42^. Most of the synaptic vesicle proteins are present in low copy numbers, the copy number of αS is high. The formation of complexes of αS and synaptic vesicle proteins in stoichiometric ratios of 1:10 or even lower seems unlikely. There is however an important exception to the low copy number found for synaptic vesicle proteins. Synaptobrevin, a v-SNARE protein involved in vesicle fusion^43^, was reported to have an average number of 70 on a single synaptic vesicle^13^. The agreement between the number of synaptobrevin and αS molecules on the vesicle surface determined by us, is striking and supports a possible role for αS in SNARE complex formation. It has been suggested that αS promotes SNARE-complex formation by a non-classical chaperone activity involving binding to synaptobrevin^6^. In this interaction the unstructured C-terminal domain of αS binds to the first 28 N-terminal residues of synaptobrevin, which are not involved in SNARE complex formation. This interaction is thought to catalyze the formation of SNARE complexes by presenting the vesicle bound synaptobrevin in the optimal conformation to interact with the plasma membrane bound SNAREs. We were not only able to confirm the interaction between αS and the soluble part of synaptobrevin, but using microscale thermophoresis we were able to determine the equilibrium dissociation constant *K*_*d*_ of the αS – synaptobrevin complex (see Supplementary Fig. 9). The micromolar binding affinity and observed αS surface concentration are in agreement with a functional biomolecular interaction^41^,^42^.

Based on the total of approximately 100 αS proteins per vesicle, the observed direct interaction of αS with the soluble part of synaptobrevin, and the ability of αS to modulate the physical properties of membranes reported in the literature^8^,^9^,^10^,^11^,^12^ we hypothesize a dual purpose of membrane bound αS. αS may both modulate the membrane’s physical properties and interact with the SNARE protein synaptobrevin. Since interactions limit conformational freedom of the unstructured C-terminal domain of αS, the formation of a αS-synaptobrevin complex may not only result in a presentation of this v-SNARE to its t-SNARE partner but also reduce the steric pressure exerted by αS. We hypothesize that the interaction between αS and synaptobrevin is regulated by a third, yet unknown, factor. Switching on the interaction between αS and synaptobrevin with this factor, may not only result in the presentation of synaptobrevin, but also cause stabilization of the disordered part of αS resulting in a decrease of steric pressure and hence possible destabilization of the vesicle. The latter two events facilitate interaction between vesicle-SNAREs and target-SNAREs which finally results in the release of synaptic vesicle content.

## Methods

### Primary neuronal cells

The extraction and culturing of primary neurons was performed as described elsewhere^44^. In short, cells were obtained from newborn (P1) Wistar rat pups. Both (cortical) cerebral hemispheres were isolated in a sterile environment, minced and trypsinized. The minced hemispheres were dissociated by trituration after which the cells were plated on polyethylenimine coated coverslips (Sigma-Aldrich, USA) to 60% density. After 2 hours, adhered cells were washed with DMEM (Invitrogen, USA) and cultured in 900 μl serum and antibiotics-free R 12 medium at 37 °C with 5% CO2 45. All research involving animals has been conducted according to Dutch law (as stated in “Wet op de dierproeven”), and approved by DEC, the Dutch Animal Use Committee.

### Construct design for αS-GFP DNA plasmid

For construction of human αS-GFP plasmid, the complete human αS cDNA was obtained from pT7-7 human αS via a PCR reaction^46^. For this, a forward primer 5′-GA**AGATCT**AAG**ATG**GATGTATTCATGAAAGGAC-3′ and reverse primer 5′-GA**AGATCTCCG**GCTTCAGGTTCGTAGTCTTG-3′ were used on a pT7-7 human-αS template. The reverse primer was designed in such a way that the natural human αS stop codon was removed. Following digestion with *Bgl*II, the human αS cDNA was isolated and ligated into the *Bgl*II and *Bam*HI sites of pEGFP-N1 plasmid. The sequence of the construct was confirmed by DNA sequencing analysis.

### Cell culture, transfection and selection of SH-SY5Y cells

The culturing, differentiation, and transfection of SH-SY5Y cells was performed as described elsewhere^47^. In short, SH-SY5Y cells were grown in proliferation medium GlutaMAX™ supplemented with 10% heat inactivated FBS, 1% non-essential amino acids, 10 mM HEPES buffer, and 1% Penicillin/Streptomycin, all obtained from Gibco^®^. For differentiation, we seeded SH-SY5Y cells to 60% confluency and induced differentiation by adding starvation medium containing 1% FBS and 10 μM retinoic acid for 3 days. All chemicals were obtained from Invitrogen, USA if not indicated otherwise.

For transfection, SH-SY5Y cells were seeded, grown until 30–50% confluency, and transfected with αS-GFP. DNA (250 ng/cm2) was diluted in Opti-MEM in reduced Serum medium (GIBCO^®^) including Lipofectamine^®^ LTX Reagent with PLUS™ Reagent. The mix was incubated for 5 minutes at room temperature before adding Lipofectamine LTX. For every 250 ng of DNA, 0.5 μl of Lipofectamine LTX and 0.19 μl of Lipofectamine PLUS reagent were mixed, according to manufacturer’s protocol. After Lipofectamine LTX addition, a 30 minute incubation at room temperature was performed. The medium of the cells was changed and the DNA, Lipofectamine LTX, and Lipofectamine^®^ LTX Reagent with PLUS™ Reagent mix were added. After one day, the medium was changed to proliferation medium. Two days after transfection, cells were trypsinized and re-seeded in conditioned medium. The next day, G418 (500 μg/ml) was added and cells were grown in G418 supplemented conditioned medium until selection by fluorescence-activated cell sorting (FACS). The αS-GFP positive cells were expanded in culture dishes and stocks were stored in liquid nitrogen.

### Immunocytochemistry and cell staining

Cell samples were washed with PBS and fixed in 3.7% paraformaldehyde/PBS solution for 30 min at room temperature (RT). For immunolabeling, primary neurons were permeabilized with 0.3% Triton X-100 and 0.1% BSA in PBS for 2 hours at RT. The permeabilization step for differentiated SH-SY5Y cells was done for 5 min at RT. Autofluorescence was quenched with 50 mM NH_4_Cl in PBS for 15 min at RT. Aspecific binding sites were blocked by incubation of the sample with goat serum dilution buffer (16% goat serum, 0.3% Triton X-100, 0.3 M NaCl in PBS) for 30 minutes at RT. Thereafter, primary antibodies (Table S1) were diluted 1:200 in goat serum dilution buffer and incubated with the sample at RT for 1 hour. Then cells were washed with 0.3% Triton X-100 and 0.1% BSA in PBS 3 times, each time for 5 min at RT, and the appropriate secondary antibodies (Table S1) were diluted 1:200 in 0.3% Triton X-100 and 0.1% BSA in PBS and incubated with the sample for 1 hour at RT. For actin staining, cells were incubated with 2 U/ml phalloidin-Alexa Fluor^®^488 (Invitrogen, USA) in PBS for 15 minutes. Nuclear counterstaining was performed by incubation in 300 nM 4′,6-diamidino-2-phenylindole (DAPI) in PBS for 10 minutes. After washing with PBS, samples were mounted with mounting medium (ibidi, Germany).

To label membranes, we used the membrane marker wheat germ agglutinin (WGA) tagged with the fluorophore AlexaFluor^®^647 (AF647) (Invitrogen, USA). WGA is a lectin protein that binds to N-acetyl-D-glucosamine and sialic acid on cell membranes^48^. For WGA-AF647 labeling, living differentiated GFP-SH-SY5Y cells were incubated with 5.0 μg/mL WGA-AF647 in medium for 10 minutes to 6 hours. Subsequently, cells were fixed and imaged as described above. Although previous reports^49^,^50^ suggest that incubation with WGA-AF647 might induce apoptosis, we did not observe any detectable increased apoptosis associated with either the amount of WGA-AF647 or incubation time in our experiments.

### Confocal fluorescence microscopy

Confocal laser scanning microscopy images were obtained using acommercial Nikon A1 confocal microscope with a 100 x oil immersion objective (NA = 1.4, Nikon, Japan) and laser sources of 405 nm, 488 nm, and 561 nm. Emission was detected using appropriate dichroic mirrors and filter sets.

### Single-molecule imaging and photo-bleaching experiments

The photo-bleaching experiments were performed using an ultra sensitive custom-built inverted confocal microscope, described in detail elsewhere^23^. In short, as excitation source, we used a pulsed diode laser operating at 485 nm at a repetition rate of 20 MHz (LDH-D-C-485, Picoquant, Germany). An epi-illumination configuration was used, i.e., the illumination and emission are collected through the same microscope objective (UPLSAPO 60XW, 60X, 1.2NA, Olympus). The remaining excitation light in the detection path was suppressed with a long pass filter (RazorEdge^®^, 488 nm, Semrock, USA) and a notch filter (StopLine^®^, 488/14 nm, Semrock, USA). An additional band pass filter for the green channel (BrightLine^®^, 550/88 nm, Semrock, USA) and a long pass filter for the red channel (RazorEdge^®^, 664 nm, Semrock, USA) were used. The emission was spatially filtered using a 15 μm pinhole and was subsequently focused onto a single photon avalanche diode (SPCM-APQR-16, PerkinElmer), connected to a photon counting module (PicoHarp300, Picoquant, Germany). The sample was first scanned at a high scanning speed, 1 ms per pixel, and low excitation power, ~80 W/cm^2^, to prevent dye bleaching. In the initial scan, we located individual αS-GFP puncta in PFA-fixed cells, localized them in the focus of the objective, and sequentially collected fluorescence intensity time traces from distinct αS-GFP puncta. To obtain the intensity time trace we used higher excitation powers, ~800 W/cm^2^, to make sure that all fluorophores of a punctum photo-bleached within 1 minute.

After acquiring the photo-bleaching intensity time trace for the punctum (see Supplementary Fig. 8A), a previously reported data analysis has been used^24^. In short, a forward-backward non-linear filtering was used to de-noise the trace. Then, a power spectrum of the pairwise difference distribution function for each of the traces was obtained (see Supplementary Fig. 8B). The unitary step size – average effective emitted photon count from a single GFP until it photo-bleaches in each punctum – has been determined based on the power spectrum following the method by *Leake* and coworkers (**Nature** *2006* 355–358), where the first significant peak against the background noise gives the step size. In the assignment of the step size, the presence of the second order peak was included in the selection criteria. Using the determined unitary step size, the total number of emitters per punctum was calculated.

Intensity profiles of the puncta were determined with Symphotime 64 (Picoquant, Germany) by applying a script that applies a Gaussian fit and calculates the Full Width Half Maximum (FWHM).

### Calculating co-localization percentage between αS-GFP puncta and membrane marker

Two images were made sequentially using the 485 nm and 640 nm excitation of the single-molecule sensitive microscope. To quantify the co-localization between αS-GFP puncta and the membrane marker WGA, the ratio of puncta in the green channel (485 excitation) which co-localized with signal in the red channel (640 excitation), to all puncta in the green channel, was calculated. Then, each punctum that appeared in the red channel (485 excitation) image, but not in the green channel, was photo-bleached using the 640 nm laser light. After photo-bleaching, the number of puncta that additionally appeared in the green channel of 485 excitation were added to the numerator and denominator of the earlier ratio calculation, and the co-localization percentage was re-calculated.

## Additional Information

**How to cite this article**: Fakhree, M. A. A. *et al*. The number of α-synuclein proteins per vesicle gives insights into its physiological function. *Sci. Rep.* **6**, 30658; doi: 10.1038/srep30658 (2016).

## Supplementary Material

Supplementary Information
