# Supplementary material for: The number of α-synuclein proteins per vesicle gives insights into its physiological function
Source: Sci Rep. 2016 Aug 1;6:30658. doi: 10.1038/srep30658 (PMC4967914; doi:10.1038/srep30658)
Supplement: Supplementary Information [file srep30658-s1.doc]

**Supplementary Information:**

**The number of α-synuclein proteins per vesicle gives insights into its physiological function**

Mohammad A.A. Fakhree^1^, Niels Zijlstra^1^, Christian C. Raiss^1^, Carolus J. Siero^1^, Heinrich Grabmayr^2^, Andreas R. Bausch^2^, Christian Blum^1^ & Mireille M.A.E. Claessens^1,*^

1: Nanobiophysics Group, MIRA and MESA+ Institutes, University of Twente, Enschede, the Netherlands

### 2: Lehrstuhl für Biophysik (E27), Technische Universität München, 85748 Garching, Germany

**Supplementary Figures:**

| 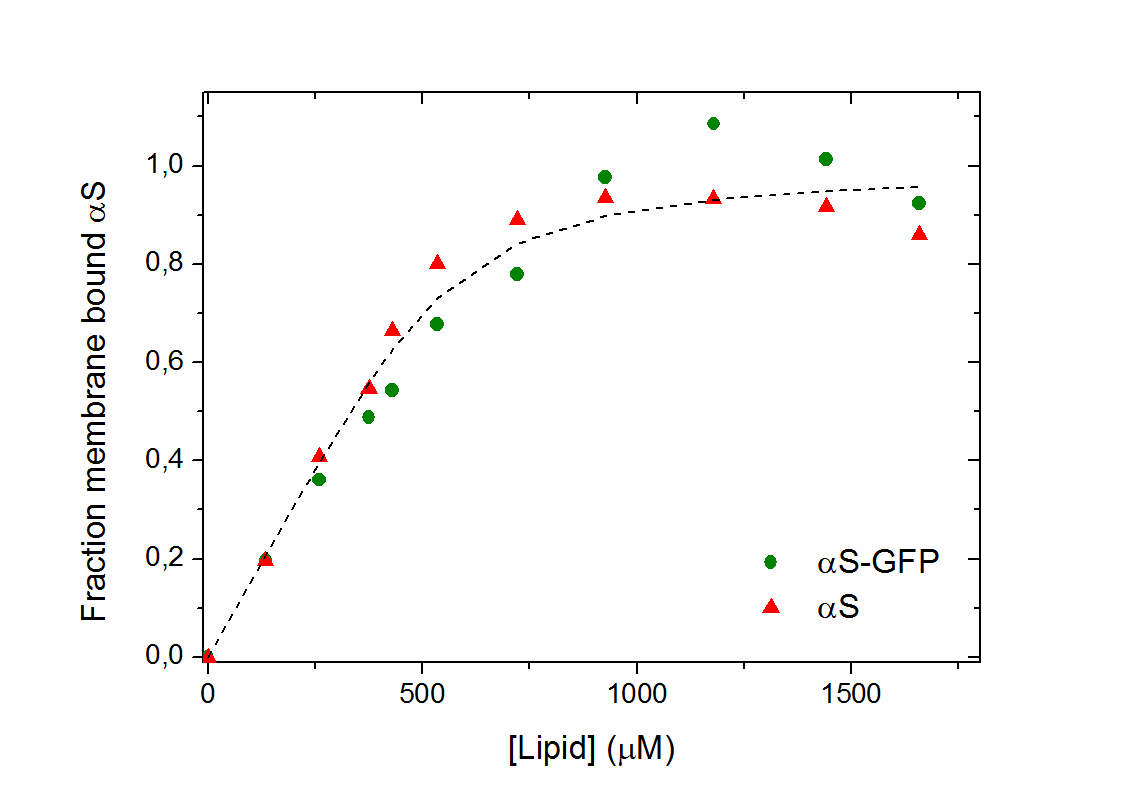 | **Figure S1.** Binding of recombinantly expressed GFP tagged (red triangles) and untagged αS (green circles) to POPC:POPS (1:1) SUVs. Membrane binding was quantified in CD spectroscopy experiments by following αS’s structural transition from a random coil in solution to a membrane bound α-helix. The bound fractions were obtained from the mean residue ellipticity at 222 nm. |
| --- | --- |
| 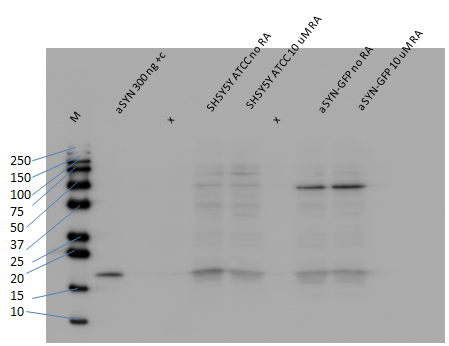 | **Figure S2.** Western blot for αS in wild type (ATCC®) and αS-GFP expressing SH-SY5Y cells in undifferentiated (no RA) and differentiated (10 µM RA) cultures. |

| 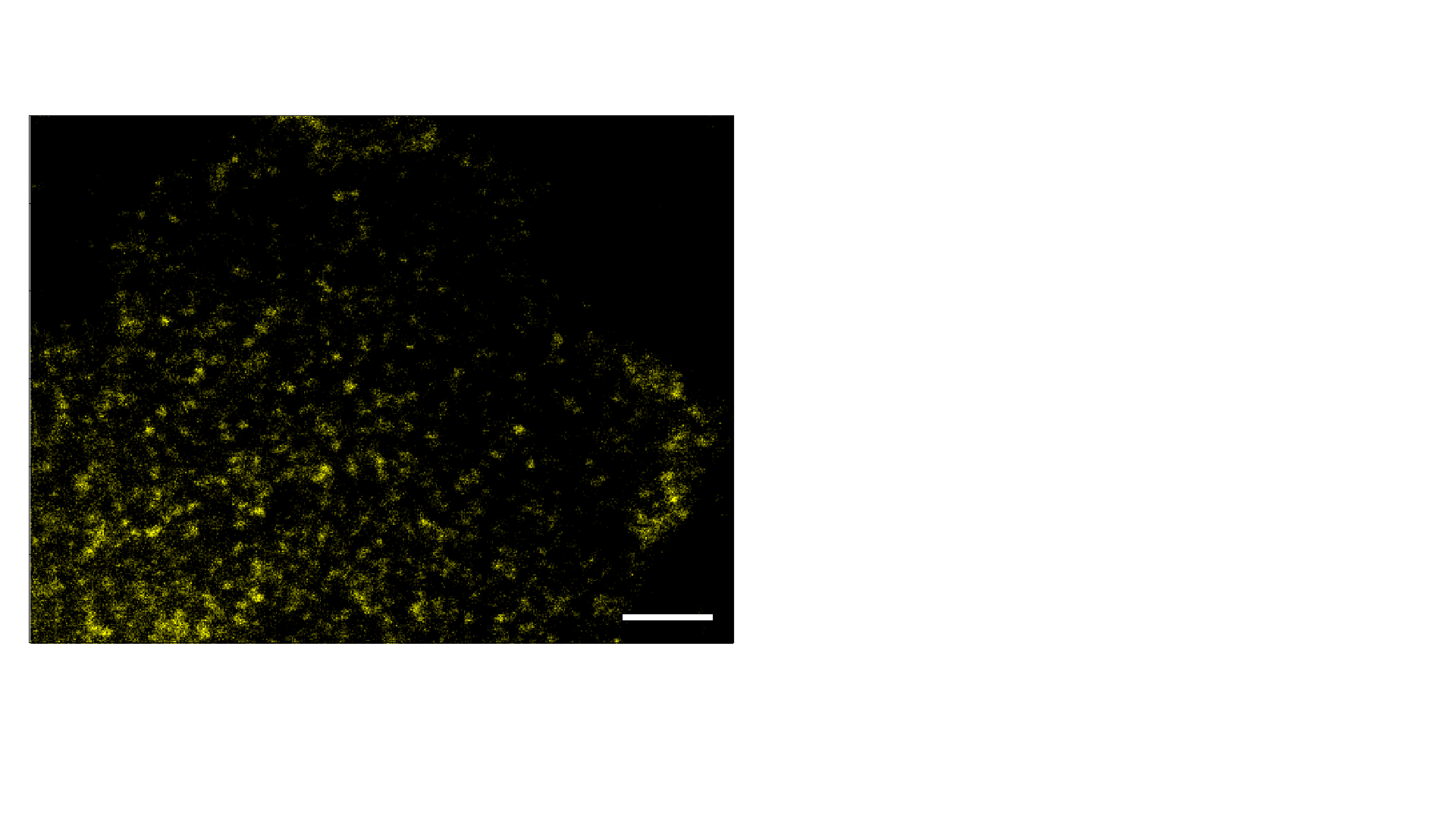 | **Figure S3.** Stimulated emission depletion (STED) super-resolution microscopy image of differentiated αS-GFP expressing SH-SY5Y cells, fixed and immunostained for αS (secondary antibody labeled with AlexaFluor®594). STED microscopy on the periphery of the cells resolves the puncta up to 80 nm, which is the resolution limit of the microscope (scale bar 1 μm). |
| --- | --- |

|  | **Figure S4.** Spectrofluorimetry of labeled WGA (5 µg/ml) in the presence of labeled αS (~3 µM) or GFP (~3 µM). The concentrations used were similar to the cell experimental conditions. After addition of the FRET acceptor to a solution of FRET donor molecules, there is no relevant reduction in donor emission intensity, or increase in acceptor emission intensity. The small reduction in donor emission intensity might be dilution effect resulting from addition of the acceptor solution. These graphs indicate that, in solution, there is no relevant FRET between WGA labeled with AlexaFluor®647 and αS labeled with AlexaFluor®488, or GFP. Since there is no FRET, we conclude there is no direct interaction between WGA and αS, or GFP. |
| --- | --- |
|  |

| 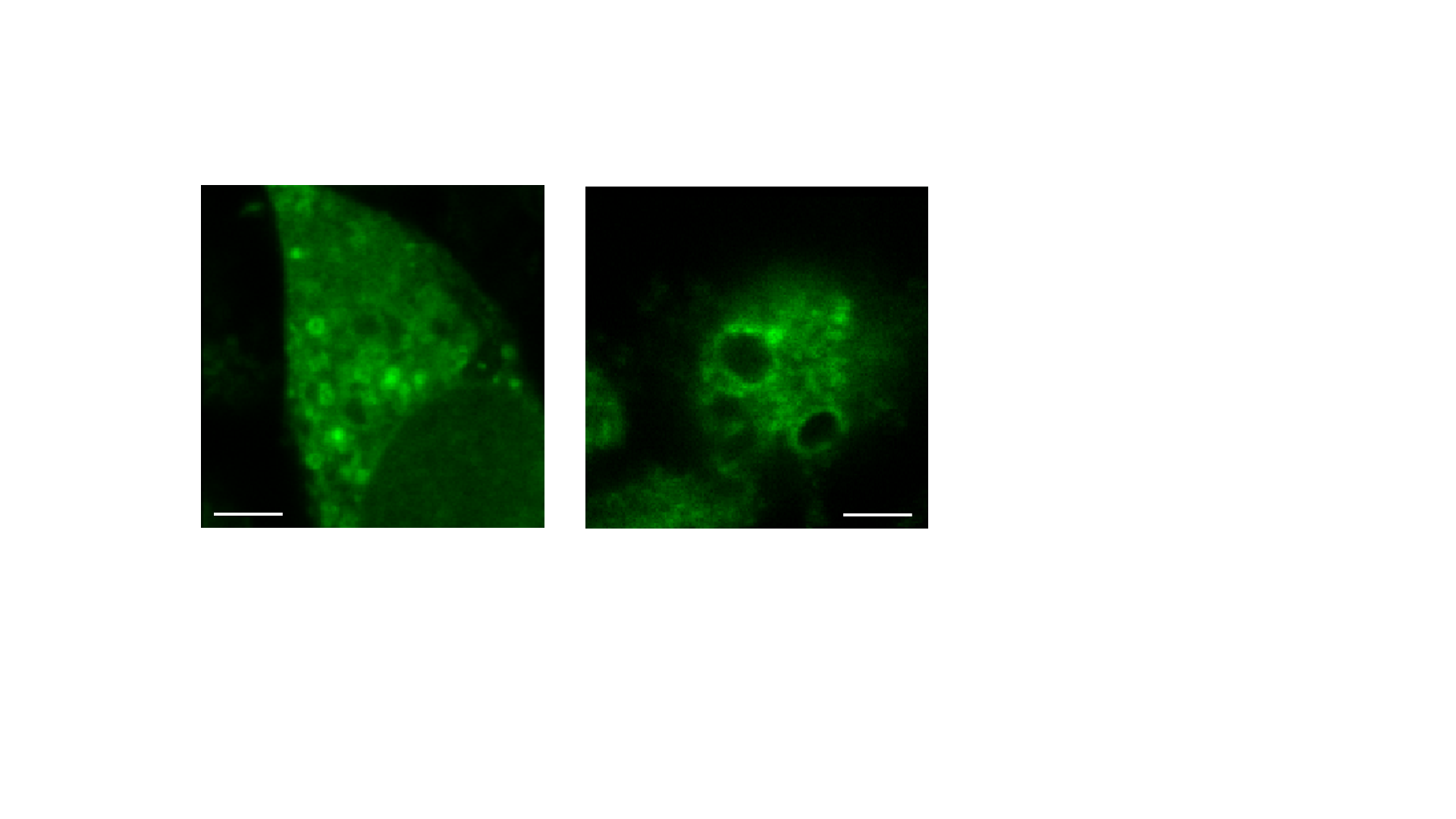 | **Figure S5.** Fluorescent signal originating from αS-GFP in differentiated SH-SY5Y cells (scale bars are 4 µm). Besides small αS-GFP vesicles which appear as diffraction limited puncta, larger vesicular structures are visible in the soma of the cells. The αS-GFP is located at the periphery of these large vesicular structures. This further hints toward a localization of αS on the surface of vesicles. |
| --- | --- |

|  | **Figure S6.** **Distribution of the number of fluorophores per UV (N=71).** POPG UVs with an average radius of 50 nm doped with 0.05 % rhodamine labeled DOPE, were used as a control model system for the photo-bleaching experiments and data analysis. The calculated average number of fluorophores per UV is 50, and the experimental results obtained from analyzing 71 photo-bleaching traces shows an average number of 54 fluorophores per UV. |
| --- | --- |

|  | **Figure S7. Determined size distribution of the studied puncta (N=243).** To have a defined selection of vesicles that are diffraction limited in size, we only studied puncta with radius of less than 350 nm, based on a Gaussian fit to the measured intensity profile of the punctum. |
| --- | --- |

|  | **Figure S8A. Photo-bleaching trace for αS-GFPs on a vesicle.** αS-GFPs on single vesicles observed in SH-SY5Y cells, was photo-bleached using high intensity excitation light (485nm, 800 W/cm^2^). Photo-bleaching was followed in time until there was no significant reduction in fluorescence intensity anymore. Because of the high intensity bleaching light, generally after 60 seconds there was no further reduction in fluorescence intensity. |
| --- | --- |

| 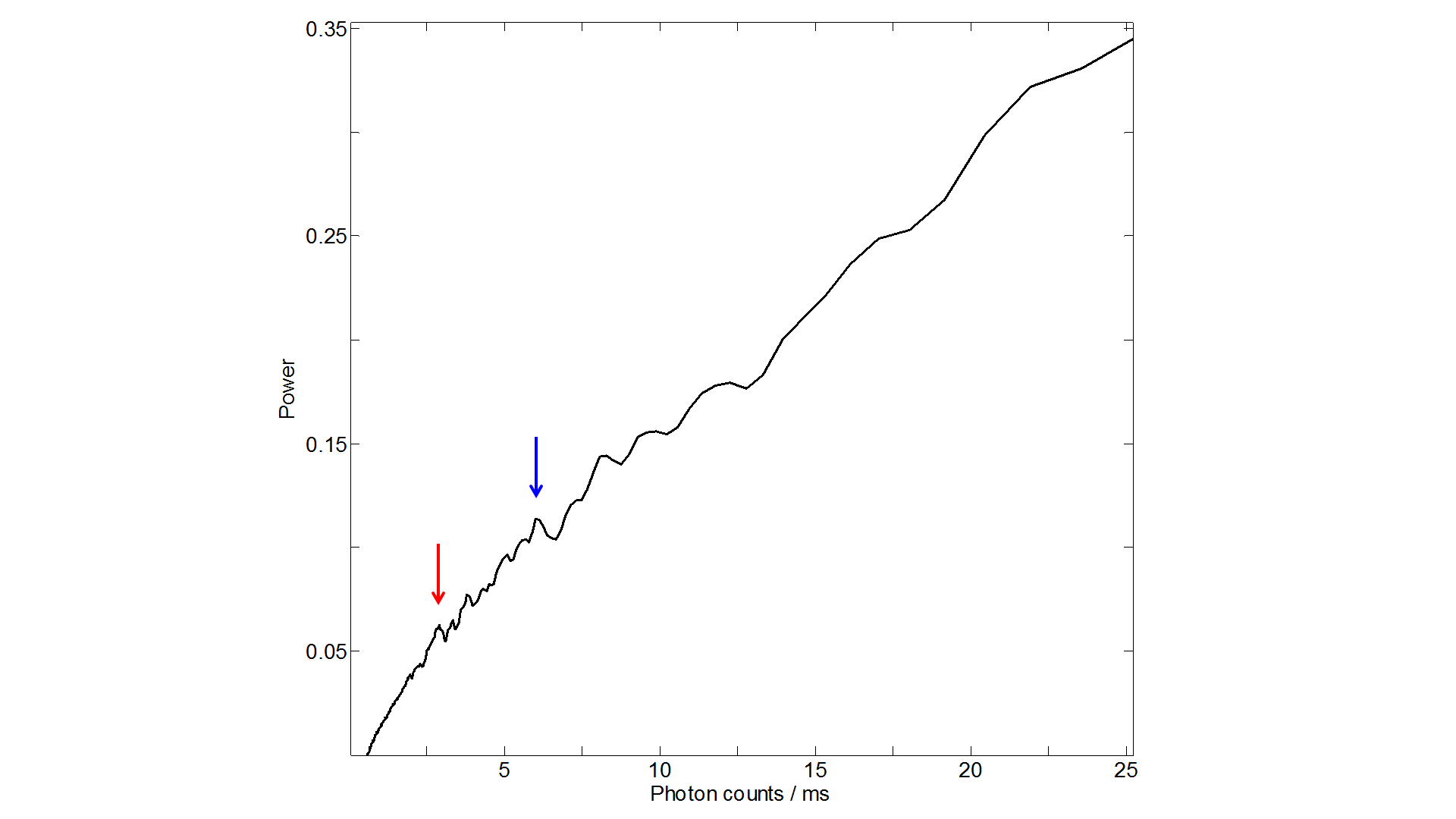 | **Figure S8B.** **Power spectrum obtained from a single photo-bleaching trace.** From the photo-bleaching traces (Fig S7A), power spectra were obtained of the pairwise difference distribution of fluorescence intensity. In the power spectrum, the step size associated with the photo-bleaching of a single αS-GFP was assigned to the first significant peak against the background noise (indicated by red arrow). In the assignment of the step size, the presence of the second order peak (indicated by blue arrow) was included in the selection criteria. |
| --- | --- |

| 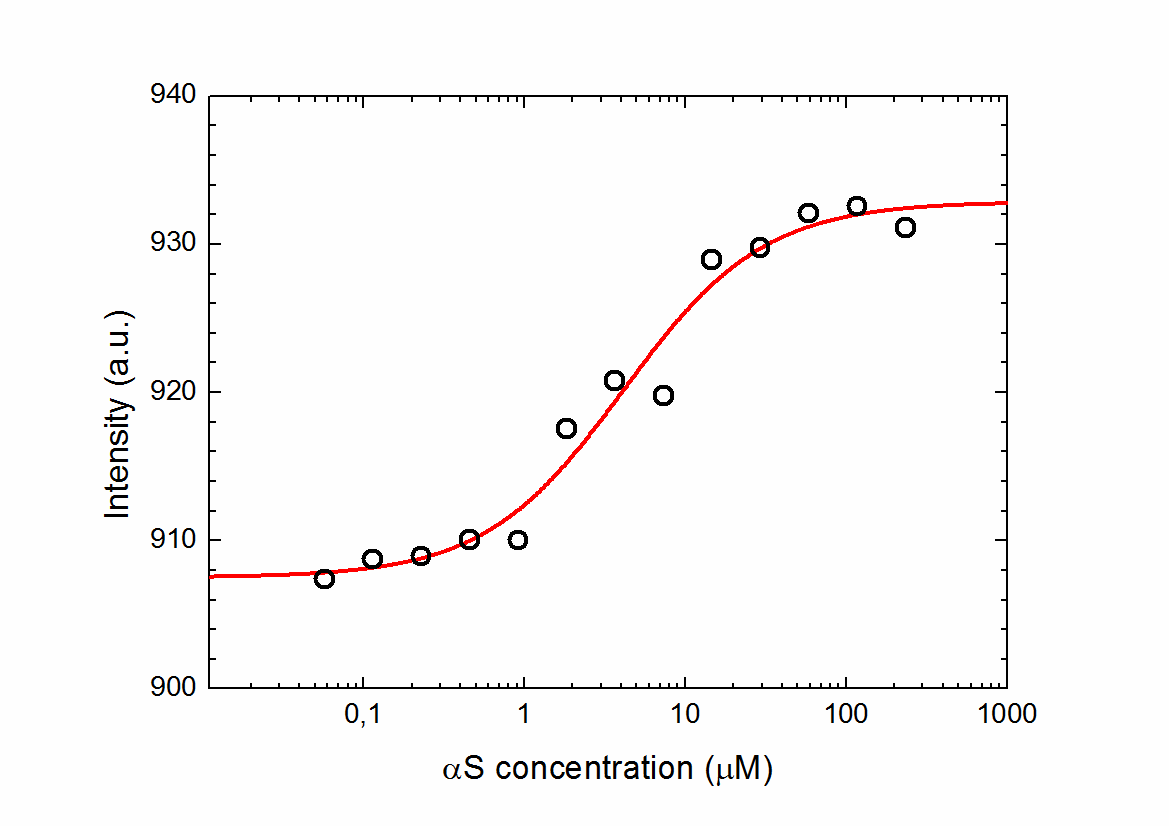 | **Figure S9**. Binding of recombinantly expressed αS to the soluble part of the SNARE protein synaptobrevin. Complex formation was quantified in a microscale thermophoresis experiment at a synaptobrevin concentration of 30 nM. The data points represent the average of 3 measurements. The red line represents a fit through the data assuming a bimolecular binding with an equilibrium dissociation constant *K_d_* = 4 µM. |
| --- | --- |

**Table S1**. List of the antibodies used in this study

| **Primary antibody** | | | |
| --- | --- | --- | --- |
| **protein** | **epitope** | **host species** | **source** |
| αS | 15-123 | rat | BD biosciences |
| αS | 121-125 | mouse | Santa Cruz |
|  | | | |
| **Secondary antibody** | | | |
| **host** | **anti** | **conjugated dye** | **source** |
| goat | rat | AlexaFluor®594 | Invitrogen |
| goat | mouse | AlexaFluor®555 | Invitrogen |

**Supplementary Methods:**

**Recombinant αS-GFP production.** An αS-GFP producing vector was prepared by insertion of the WT αS coding DNA in the pRSETa-EGFP vector[^1^](#_ENREF_1). The stop codon was removed from the WT αS in the pT7 plasmid[^2^](#_ENREF_2), NheI overhang was created with DNA linkers. pRSETA-EGFP and the α-synculein with NheI overhang were cut with NheI and BglII. αS was ligated into the pRSETA-GFP, creating a 6His-syn-GFP producing vector. The vector was transformed into BL21(DE3)pLysS. An overnight culture (37 °C, 200 rpm) was diluted 100x and 1mM IPTG (Thermo Fisher Scientific Inc., US) was added at an OD_600nm_ of 0.6 to induce the expression of the protein. The temperature was decreased to 20 °C during the expression. After 18 hours bacteria were pelleted by centrifuging 10 minutes at 6000g. The bacteria were lysed by 6 freeze-thaw cycles in liquid nitrogen and water and resuspended in a solution of 50 mM NaH2PO4, 300 mM NaCl, 10 mM imidazole. The cells were sonicated on ice for 10 seconds (Branson 250 sonicator) before centrifugation for 20 minutes at 10000g. Supernatant was incubated with Ni-NTA agarose (Qiagen) for 1 hour at 4 °C while stirring. This mixture was poured in an empty PD-10 column (GE) and washed twice with a solution of 50 mM NaH2PO4, 300 mM NaCl, 20 mM imidazole. Protein was eluted with a solution of 50 mM NaH2PO4, 300 mM NaCl, 250 mM imidazole. The buffer was exchanged for 10 mM Tris pH 7.4, 50 mM NaCl on a PD-10 column (GE). The protein concentration was determined using Nanodrop ND-1000 spectrophotometer (Thermo Fisher Scientific Inc., US) assuming a molar extinction coefficient of 56000 M^-1^cm^-1^ for the GFP at 507 nm.

**STED microscopy.** Stimulated emission depletion (STED) microscopy[^3^](#_ENREF_3) was employed for sub-diffraction limit resolution fluorescence imaging on a custom-made setup. The system’s implementation is based on a supercontinuum laser source, and similar to the setup described elsewhere [^4^](#_ENREF_4). It is capable of acquiring one channel with confocal and two channels with STED resolution quasi-simultaneously. The supercontinuum laser source was a SC450-PP-HE system running at 1 MHz (Fianium Ltd, UK). For beam-scanning, we used a YANUS IV scan head from Till Photonics, Germany. The objective was a Leica 100x/1.4. For imaging green fluorescent protein (GFP), Alexa Fluor® 594 and Alexa Fluor® 647, we used excitation/emission wavelengths of 488±3 nm/520±14 nm, 586±7 nm/624±40 nm and 637±5 nm/ 685±20 nm, respectively, using optical filters (AHF, Germany). The STED wavelengths for Alexa Fluor® 594 and Alexa Fluor® 647 were set to 720±10 nm, and 750±10 nm, respectively. Beam powers for acquisition were 1 - 5 µW for the excitation beams, as measured in front of the objective. STED beam powers amounted to 1 - 2 mW. To reduce crosstalk, pulses for various channels were separated in time by varying optical path lengths. A home-built electronic gating device transmitted detector signals occurring at the correct time to the acquisition hardware, and rejected crosstalk signals occurring at other times. Dichroic mirrors and filters were purchased from AHF, Germany.

**POPG UV model system.** 1-palmitoyl-2-oleoyl-sn-glycero-3-phospho (1’-rac-glycerol) (sodium salt) (POPG) and Rhodamine-6-G labeled 1,2-dioleoyl-sn-glycero-3-phosphoethanolamine (Rhod-DOPE) lipids were purchased from Avanti Polar Lipids (Alabaster, AL) and were used without any further purification. Lipid mixtures were prepared from a 10 mg/ml stock solutions in chloroform. The labeled lipid, Rhod-DOPE, was added to the POPG solution to end up with a mixture containing 0.05% of labeled lipids. To prepare Unilamellar Vesicles (UVs) the membrane extrusion method was used following the protocol of the provider (Avanti Polar Lipids). In short, a mixture of lipids dissolved in chloroform, was dried using a nitrogen stream. After drying of the lipids, an appropriate volume of phosphate buffer saline (PBS) was added to the dry lipid layer to reach final lipid concentration of 500 µM. This suspension of lipids, was mixed and subjected to 12 cycles of freeze/thawing using liquid nitrogen and 37 °C water. The thus obtained lipid dispersions were filtered at least 11 times using 1000µl MICROLITER syringes through an Avanti Polar Lipids mini Extruder containing a Nuclepore track-Etch Membrane with a pore size of 0.1 µm. This resulted in UVs with diameter of approximately 100 nm. The UV sample was diluted 1:200 in PBS and used in single-molecule microscopy setup for photo-bleaching experiments.

We calculated the average number of lipids and labels per UV as follows:

The area per monolayer of a vesicle with a radius of 50 nm equals
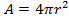
= 31415 nm^2^. This corresponds to an area of ~62830 nm^2^ for the lipid bilayer of one UV. The head group size of POPG is estimated to be 0.628 nm^2^ [^5^](#_ENREF_5), and therefore a single UV consists of approximately 62830/0.628 = 100000 lipids. With 0.05 % of labeled lipids, this corresponds to 50 labeled lipid per UV.

**POPC:POPS SUV model system.** Stock solutions of 1-palmitoyl-2-oleoyl-sn-glycero-3-phosphocholine (POPC) and 1-palmitoyl-2-oleoyl-sn-glycero-3-phospho-L-serine (POPS) were purchased from (Alabaster, AL) and were used without any further purification. Stock solutions of POPC and POPS were mixed in a 1:1 molar ratio, dried under a stream of nitrogen and placed under vacuum for 1 hour. The dried lipid films were subsequently rehydrated in a 10 mM Tris, 100 mM KCl solution and vortexed for 4 minutes. Small unilamellar vesicles (SUVs) were prepared by sonicating the rehydrated liposome solution for 40 minutes using a Branson tip sonicator. Thereafter SUVs were centrifuged at 13200 rpm to remove possible residue from the sonicator probe. The SUVs were stored at 4 °C and used for experiments within 2 days.

**Circular dichroism (CD) spectroscopy.** CD spectra were obtained on a Jasco J-175 spectropolarimeter. POPC:POPS (1:1) SUVs were titrated to a protein concentration of 3 µM. Upon binding to vesicles, the initially unstructured (random coil) αS undergoes a structural transition to an α-helix. Membrane binding can therefore be visualized by following this structural transition. For this purpose we followed the change in absorbance at 222 nm that is indicative of α-helix formation as a function of the lipid concentration. To obtain the bound fraction the obtained mean residue ellipticity values for were normalized assuming that the plateau MRE values represent saturation of protein binding sites on the lipid membrane. CD spectra of samples in 1 mm thick cuvettes were recorded with a step size of 1 nm and a scan speed of 10 nm/minute at room temperature.

**Recombinant synaptobrevin production.** The pET28a plasmid with a kanamycine resitence site and NHe1-Xh01 cutting sites encoding the His_6_-tagged soluble part of rat synaptobrevin B2 was a kind gift from D. Fasshouer (DNF, University of Lausanne, Switzerland). The synaptobrevin B2 protein is truncated at the C-terminus leaving only the first 96 amino-acids. The pET28a plasmid was transformed into BL21(DE3) competent cells for protein expression. A culture of the transfected bacteria was incubated at37 °C to expand the bacterial solution to an optical density of 0.6. Subsequently the T-7 promotor was induced by adding IPTG to induce the expression of protein. The temperature was decreased to 20 °C during the expression. After 16 hours bacteria were pelleted by centrifuging 10 minutes at 10000g. The bacteria were lysed and the insoluble material was separated from the soluble material by centrifugation for 30 minutes at 1500 g, 4⁰C. This solution was loaded onto a 6 ml ResourceQ column coupled to an Äkta purfier system using a loading buffer containing 500 mM NaCl, 20 mM Tris, 8 mM imidazole. The His_6_ tagged synaptobrevin protein was eluted from the ResourceQ column using a linear imidazole gradient (8-500 mM) in 500 mM NaCl, 20mM Tris, pH 8. The protein concentration was determined using Nanodrop ND-1000 spectrophotometer (Thermo Fisher Scientific Inc., US) assuming a molar extinction coefficient of 12490 M^-1^cm^-1^ for the GFP at 280 nm.

**Microscale thermophoresis.** Synaptobrevin (soluble part) was labelled using an amine-reactive labelling kit, Monolith NT™ Protein Labelling Kit BLUE-NHS (NanoTemper® Technologies GmbH, München, Germany). Labeled synaptobrevin was diluted to a concentration of 30 nM in the final solution measured and mixed with αS of different concentrations, see Figure S9. The buffer used for the microscale thermophoresis experiments consisted of 10 mM Tris pH 7.4, 0.5 mg/ml BSA, 0.05% Tween, 10 mM NaCl dissolved in MilliQ-water. Microscale thermophoresis was performed using a Monolith NT.115 MST (NanoTemper® Technologies GmbH) equipped with the Nano –BLUE/GREEN filter combinations. The data was recorded using NT Control 2.0.2.29 (NanoTemper® Technologies GmbH) and processed using NT Analysis 1.5.41 (NanoTemper® Technologies GmbH)

**Supplementary information references:**

1. Volkmer A., Subramaniam V., Birch D. J. & Jovin T. M. One- and two-photon excited fluorescence lifetimes and anisotropy decays of green fluorescent proteins. *Biophys J* **78**, 1589-1598 (2000).

2. Paleologou K. E. *et al.* Phosphorylation at Ser-129 but not the phosphomimics S129E/D inhibits the fibrillation of alpha-synuclein. *Journal of Biological Chemistry* **283**, 16895-16905 (2008).

3. Klar T. A. & Hell S. W. Subdiffraction resolution in far-field fluorescence microscopy. *Opt. lett.* **24**, 954-956 (1999).

4. Wildanger D., Rittweger E., Kastrup L. & Hell S. W. STED microscopy with a supercontinuum laser source. *Opt. Express* **16**, 9614-9621 (2008).

5. Murzyn K., Rog T. & Pasenkiewicz-Gierula M. Phosphatidylethanolamine-phosphatidylglycerol bilayer as a model of the inner bacterial membrane. *Biophys. J.* **88**, 1091-1103 (2005).
